# Time-Based Measurement of Personal Mite Allergen Bioaerosol Exposure over 24 Hour Periods

**DOI:** 10.1371/journal.pone.0153414

**Published:** 2016-05-18

**Authors:** Euan R. Tovey, Damien Liu-Brennan, Frances L. Garden, Brian G. Oliver, Matthew S. Perzanowski, Guy B. Marks

**Affiliations:** 1 Woolcock Institute of Medical Research, The University of Sydney, Glebe, New South Wales, Australia; 2 South Western Sydney Clinical School, University of New South Wales, Liverpool, New South Wales, Australia; 3 Ingham Institute of Applied Medical Research, University of New South Wales, Liverpool, New South Wales, Australia; 4 School of Life Sciences, University of Technology Sydney, Broadway, New South Wales, Australia; 5 Department of Environmental Health Sciences, Mailman School of Public Health, Columbia University, New York, United States of America; Telethon Institute for Child Health Research, AUSTRALIA

## Abstract

Allergic diseases such as asthma and rhinitis are common in many countries. Globally the most common allergen associated with symptoms is produced by house dust mites. Although the bed has often been cited as the main site of exposure to mite allergens, surprisingly this has not yet been directly established by measurement due to a lack of suitable methods. Here we report on the development of novel methods to determine the pattern of personal exposure to mite allergen bioaerosols over 24-hour periods and applied this in a small field study using 10 normal adults. Air was sampled using a miniature time-based air-sampler of in-house design located close to the breathing zone of the participants, co-located with a miniature time-lapse camera. Airborne particles, drawn into the sampler at 2L/min via a narrow slot, were impacted onto the peripheral surface of a disk mounted on the hour-hand of either a 12 or 24 hour clock motor. The impaction surface was either an electret cloth, or an adhesive film; both novel for these purposes. Following a review of the time-lapse images, disks were post-hoc cut into subsamples corresponding to eight predetermined categories of indoor or outdoor location, extracted and analysed for mite allergen Der p 1 by an amplified ELISA. Allergen was detected in 57.2% of the total of 353 subsamples collected during 20 days of sampling. Exposure patterns varied over time. Higher concentrations of airborne mite allergen were typically measured in samples collected from domestic locations in the day and evening. Indoor domestic Der p 1 exposures accounted for 59.5% of total exposure, whereas total in-bed-asleep exposure, which varied 80 fold between individuals, accounted overall for 9.85% of total exposure, suggesting beds are not often the main site of exposure. This study establishes the feasibility of novel methods for determining the time-geography of personal exposure to many bioaerosols and identifies new areas for future technical development and clinical applications.

## Background

Allergic diseases are a major and increasing global health problem. Worldwide more than 300 million people have asthma, and sensitisation rates to common environmental allergens among children are approaching 40–50% [1]. This markedly affects the quality of life of these individuals and their families, and negatively impacts on the socio-economic welfare of society.

The most common allergens that people are allergic to, besides pollens, are those from domestic sources, including dust mites, cockroaches and furred animals, depending on microclimate and social factors. Despite the apparent logic of using domestic interventions to reduce exposure and thus prevent disease or reduce symptoms, success with this approach has been elusive [2]. Among the multiple possible reasons for this failure are the complexities of the roles of allergen and non-allergen exposures in the aetiology and symptoms of these diseases, including a lack of understanding about the nature and sites of allergen exposure.

Most studies that have attempted to estimate domestic allergen exposure have used the concentration of allergen measured in a settled dust sample collected by vacuuming a single site in the house as a proxy for domestic exposure. Because beds have relatively high concentrations of mite allergen in their dust compared to other domestic sites, they have often been regarded as the major source of mite exposure and a major target for interventions. However there is emerging evidence that for mite and probably other allergens, these models are over-simplified and misleading [3]. Measuring personal exposure to airborne allergens over time is challenging for a number of technical and logistic reasons.

Recent studies of real-time exposure to airborne particles using laser particle counting provide some insights into the probable ‘time-geography’ of domestic exposure to allergen bioaerosols. These demonstrate that personal indoor exposure to particles fluctuates greatly both temporally and spatially and is the complex product of many factors, including the number and source strength of multiple local reservoirs, the disturbance of these reservoirs by people or by other activities, the dispersion of such particles by airflow, thermal currents and ventilation, and the effects of particle settling [4–8].

At present there are no established methods to measure the patterns of daily personal exposure to aeroallergens or other bioaerosols, as real-time detection is not feasible. As a result we do not know when and where most inhaled exposure to mite allergens occurs. Additionally, it is apparent that significant exposure to ‘domestic allergens’ also occurs in non-domestic location such as occupational, educational and public settings [9, 10] due to the widespread dispersion of allergens to these places on clothing. This further increases the uncertainty about important sites of exposure over the day.

We recently performed the first assessment of mite aeroallergen exposure over the course of 24 hour periods [11]. In that study, subjects used a small filter-type air sampler and pump to collect continuous serial samples. During the non-sleeping period in the day and evening, the sampler was worn on the lapel and the filter was changed every 2 hours for a total of 16 hours, plus at night a single filter was collected from the head of the bed for 8 hours. Participants kept written records of their activity and attempted to collect visual records using an Apple iPod running a time-lapse application. Exposure during individual activities was derived from a mixed model analysis. This showed people and activity were the main drivers of mite exposure, which generally was low in bed. However the precision of the analysis was reduced by the frequent occurrence of multiple different activities within each 2-hour sampling frame, coupled with the variable quality of the participants’ record keeping.

In order to better determine the pattern of personal aeroallergen exposure over time, we have developed a time-based personal sampling system for allergens that allows post-hoc selection of subsamples for analysis; this is combined with an improved system for logging activities. We present preliminary observational data on this, again in the context of mite aeroallergen exposure. We also demonstrate the use of different collection surfaces which would potentially enable the method to be applied to other bioaerosols.

## Materials and Methods

### Study design

We continuously sampled the personal aeroallergen exposure of a group of normal participants over 24 hour periods using a novel time-based sampler of in-house design. We simultaneously recorded each participant’s location and activity using a miniature time-lapse camera mounted alongside the sampler. The proportion and quanta of daily mite aeroallergen exposures which was associated with different categories of locations was determined. As this study was proof-of-principle rather than hypothesis driven, the sample size was chosen within the constraints of the available budget and informed by our previous study [11].

### Setting

The samples were collected in Sydney, Australia between January and June 2014. Participants were normal, healthy adults who lived typical lifestyles in a representative range of dwellings and had no diseases that would modify their behaviour. Participants included three of the authors and the remainder were friends, colleagues or associates.

### Ethics statement

All participants provided written informed consent and retained a copy of the Participant Information Sheet. The study was approved by the University of Sydney, Human Research Ethics Committee; approval number 11392.

### Monitoring of exposure

**Fig 1:**
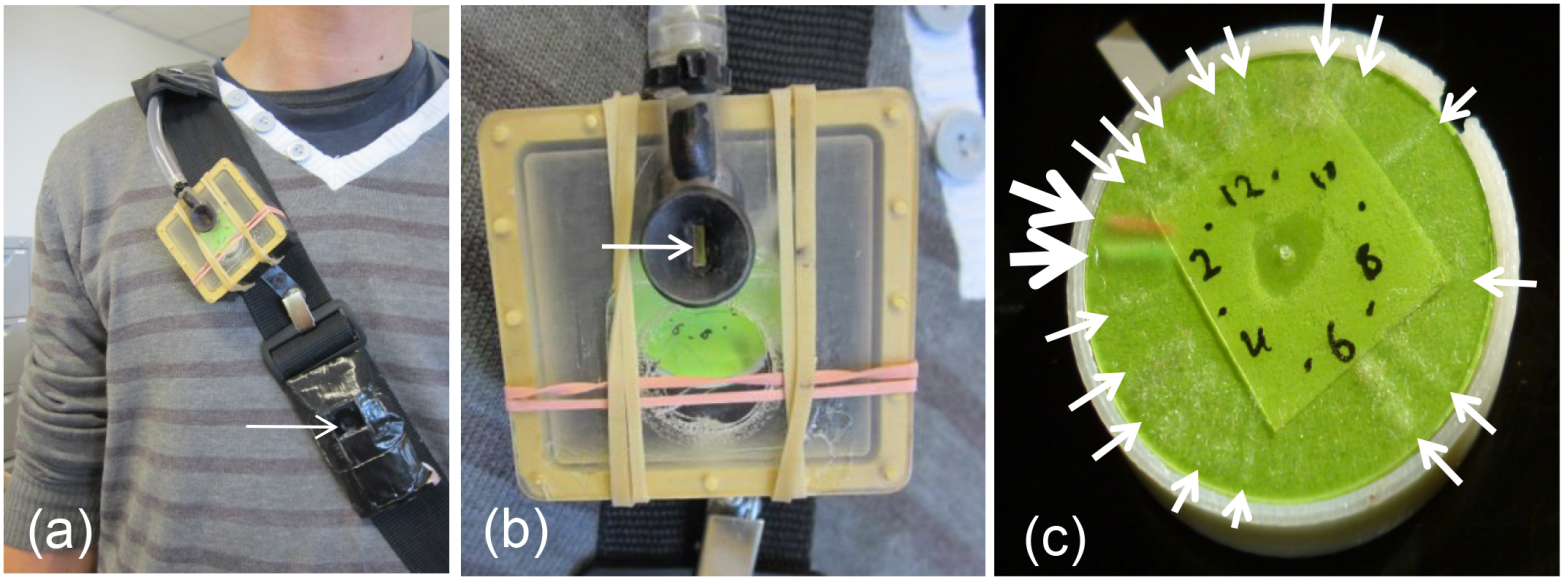
Images of the sampler and disk as used for particle collection. (A) The sampler as worn on shoulder strap of a small backpack containing the airpump; the arrow indicates the location of the time-lapse camera. (B) Close-up of the sampler; the arrow indicates the inlet. (C) An adhesive disk following collection for approximately 11.5 hours. The large arrows indicate the green and red powder used to mark the start and stop of sampling and smaller white arrows indicate visible bands of dust particles impacted during periods of high exposure. The numbers on the inner face are used to visually check the process during sampling.

The air sampler consisted of a small impaction collector of in-house design, of approximate dimensions 5 x 5 x 3 cm, shown (Fig 1). This was based around the body of a small 1.5 volt clock modified with parts made by 3D printing, using a local design company (DesignByThem, Sydney, Australia). The front cover of the clock was replaced by a faceplate containing an inlet slot as well as an air outlet; the back of the clock body was sealed to be airtight. The sampler orifice was modelled on our nasal air sampler [12] modified to have a 1x5 mm inlet slot. Particles were impacted around the outer face of a 25 mm diameter disk affixed to the hour hand shaft of the clock. Both 12 hr and 24 hour clock motors (Quartex High Torque Movement Series Part no. 101140 (12h) and Part no. 101070 (24h)) were used. The 50% cut point was calculated to be 3.5 μm, based on standard formulae [13]. For collection, the sampler and camera were mounted on the front diagonal shoulder strap of a small back-pack which contained the air pump (Casella Tuff, Bedford, UK) running at 2L/min. The pump itself was enclosed in a plastic lunchbox lined with acoustic foam to reduce noise. The 3D printer files for this design and for a subsequent modified sampler design are provided in S1 3D File.

#### Sampling surface

Two different collection/impaction surfaces were used; one was a single layer of a thin electrostatic cloth (electret, EWE50, Vilene, Japan), the other was the adhesive surface of the film used to protect the screens of i-Phones (Daiso, Japan) and similar screen-based smart devices. The electret was fixed to overhead-projector film using double-sided tape and 25 mm diameter disks cut using a rotary paper cutter. These disks was then mounted, again using double sided tape, to the plain side of disks cut from Full Adhesive Post-it notes (3M, Kentucky, USA) so the Post-it adhesive bound reversibly to the 3D-printed disk mounted on the clock hour hand. The i-Phone adhesive (revealed by removing the protective film prior to use) was also mounted using double sided tape to the plane side of full surface adhesive Post-it notes, which were then cut into disks as above, such that the Post-it adhesive bound reversibly to the 3-D printed disks. The 3D printed disks for electret and i-Phone adhesives were of different thicknesses to maintain a distance of 1.25–1.5 mm between the jet outlet and the impaction surface.

#### Sampler efficiency

The collection efficiency of the sampler and collection surface was estimated in two ways. For 35 sets (each set being a single disk of sub-samples) of samples, an IOM sampling head was modified and mounted in series directly behind the sampler outlet, in line with the pump. The IOM head contained a filter and was analysed for allergen as previously described [11]. In a subset of 6 of these sets, the internal wall surfaces of the sampler and the inlet nozzle of the sampler were also swabbed with a swab made of electret and analysed for allergen as described.

#### Sample collection

The participants wore the small back pack containing the sampler and pump during the non-sleeping hours with the sampler positioned near the collar. At night when sleeping they placed the backpack over the corner of bed head with the sampler inlet positioned above the pillow; the typical distance from the head was 30–50 cm. Where this was not possible due to the design of some beds, the backpack was placed beside the bed alongside the pillow, with the sampler facing and as close as possible to the participant. Where the 12-hour motor was used, the participant changed the disk, after approximately 11.5 hours of collection, when the 24-hour motor was used, this was not required and sampling continued for close to 24 hours.

The start and finish times of each sampling were marked by aerosolising a green or red powder (indicating start and finish times respectively) in the proximity of the sampler orifice, using a small powder blower (Teijin, Japan). The powder was made by sanding the surface of blocks of water-color paints.

### Monitoring of activity

A small camera (LyfeShot, Blynk, Acton, MA, USA) was mounted below the sampler on the shoulder strap. This was set to take pictures of the surroundings at 15 or 30 second intervals. Participants were advised about issues of privacy and propriety and provided with an adhesive cover for the lens to use when social situations precluded collecting images of themselves or others. The camera was turned off while the participant was in bed.

#### Daily Activities

After sampling, the time-lapse images were viewed using the camera’s bundled software and the type and times of different activities and locations were identified and recorded on an Excel spreadsheet. The different activities were summarised, post-hoc, into 8 categories; five were ‘indoor’, two were ‘in-transit’ and one was ‘outdoor’. The ‘indoor’ categories covered (1), home–in bed sleeping at night, (2) home—in the bedroom but not sleeping; for example reading during the day, watching TV, working on a computer, (3) home–in the living room, kitchen or other family area, (4) occupational workplace, and (5) public building. The ‘in transit’ categories covered: (6) public transport (train / bus / ferry / taxi / other), and (7) private vehicles (car). The ‘outdoor’ category (8) covered any times outdoors, including when walking and cycling.

#### Selection and analysis of individual samples

Information on the time and category of activities were loaded into an excel spreadsheet, where the total time was approximately either 12 or 24 hours depending on which clock motor was used. A pie chart was constructed where each wedge of the pie chart represented a different subsample or activity. The smallest wedge that was practical to slice was ~1.5–2 mm distance at the circumference of the disk, equating to a sampling time of approximately 30 minutes using the 24 hour clock and 15 minutes using the 12 hour clock. For samples collected during the day, the aim was to have a single wedge for each activity. Where several activities occurred over a short time frame, the sub-sample contained more than one category of activity. The portion of the disk corresponding to the total time in bed was divided into four sections consisting of the first 30 minutes starting from getting into bed, the last 30 minutes prior to getting out of bed, and the remaining middle portion was divided into two equal halves equating to approximately 3 hours each, varying for each participant.

The sample disk was overlaid on the printed pie chart and used as a guide to cut the wedges of the collection surface using small scissors which were cleaned between each use. Most of the wedges were extracted for 2 hours in 150 μl of 0.2% BSA in PBS-Tw (0.5%); in some earlier experiments 300 μl was used. Samples were assayed immediately without storage.

#### Allergen assay

Samples were analysed using a commercial ELISA assay for mite allergen Der p 1, as modified by us [14]. The limit of detection was 9.8 pg Der p 1/ml. In order to detect the small quantities of allergen involved, extraction was performed in atypically small volumes and assays were performed without duplicates. Previous in-house ELISA results had demonstrated that 98.04% of duplicate samples above the minimum detection limit have a CV% of <10%, and 84.4% had a CV% of <5%. Samples falling below the limit of detection in the assay were attributed with a nominal value of half the detection limit (4.7 pg/ml) to allow calculations using log values, unless otherwise specified.

### Analysis of data

The raw data for subject ID, collection surface, quantities of allergen, sample times, and activities, along with data used to estimate sampling efficiencies and the data dictionary are provided in S1 Table.

#### Patterns of exposure of the participants

The quantity of allergen in each sample was expressed as amounts (pg) /sample and as average rate of exposure (pg/m^3^) over time. Geometric means (+/- 95% confidence intervals) of the rates of exposure for each activity were calculated using mixed model regression in which subjects were assigned random intercepts and activities were fixed effects. The relative ratio for each activity was calculated as the geometric mean quantity of allergen for each activity as a function of the geometric mean for all the other activities (+/- 95% confidence intervals and significance). The percent contribution to exposure of the different activities could not be calculated in the regression model; instead this was estimated as the sum of the quantities of exposure for all samples associated with that activity, expressed as a percentage of the sum of the quantities for all activities for the total set of samples. Similarly, the percentage of time for each activity was calculated as the sum of periods involving that activity, as a percentage of the total time of all activities.

#### Sampling efficiencies

The overall collection efficiency of the sampler was calculated as the sum on the set of segments of the disk as a percentage of the total amount of allergen (segments, internal walls, nozzle and back-filter).

Where the collection by the electret or i-Phone adhesive collection surfaces were determined, the sum of the disk segments was expressed as a percentage of the sum of the segments plus the amount collected on the back filter. For both of these determinations, a value of zero was used for samples below the detection limit of the assay, as attributing a nominal value of 4.9 pg/ml to each segment where multiple segments were involved, distorted the apparent efficiency. The efficiencies for the two sampling surfaces were compared using a Wilcoxon-Mann-Whitney test.

## Results

In our proof-of-concept evaluations, all subjects collected samples over approximately 24 hour periods with no technical or logistical problems. In the sub-study where the overall efficiency of the sampler was estimated, the sampling surface collected a geometric mean 61.5% of the total allergen aspirated, with 16.3% being lost on the inlet orifice, and 14.6% to the internal walls; losses to a back filter were mostly below the detection limit. In 35 sets of samples which used one or other of the two sampling surfaces, the electret (n = 15) collected a geometric mean of 83.9% of the total allergen while the i-Phone adhesive (n = 20) collected a geometric mean of 84.7% of the total allergen collected on the sampler and back filter. There was no difference in the percentage of the sum of the segments plus the amount collected on the back filter between the two sampling surfaces (P = 0.5). The full results for the sampler and surface efficiency are presented in S1 Table.

In the pilot field study, 11 healthy adult participants initially participated, however one had no allergen detected in any of their samples and was removed from all further analysis. Among the 10 remaining participants, eight collected a single set of samples over the course of a single day and night, one participant collected samples over four days and one participant collected six day and night samples, plus samples over three part days. These repeated days were non-continuous.

The characteristics of the 10 participants were as follows; ages ranged from 21–66 years (mean 39.2 years), 50% were male, and seven worked in the same medical research institute. Of the 20 days on which samples were collected, 9 participants spent at least one hour of that day in this Institute, and two were spent in different occupational environments, 16 spent parts of the day at home and 10 spent part of the day in public spaces. Of the 10 participants, all cohabited with other people; eight in free-standing houses, one in a terrace-house (a townhouse in US nomenclature) and one in a two-storey apartment.

The average total sampling time for each total day and night period was 22 hrs and 56 min (range 18 hr, 51min to 24 hrs, 25 min). In the time-lapse files used to classify the activities for each person, there were on average 14.15 (range of 9 to 18) activity-based segments for the day and evening, plus the additional four samples for each participant while in bed. The average duration of the day-time segments was 58.9 minutes. In total 353 samples were analysed; of these n = 143 were collected on electret cloth and n = 210 on adhesive film. The number of activities per sample varied; 73.6% of samples had one activity, 22.8% had two, 4.2% has three and one sample (0.3%) had four activities. In total there were 463 occurrences of activities, categories 1–8, among the 353 samples.

Of the 353 samples, 202 (57.2%) had measureable Der p 1 (that is, >1.47 pg Der p 1/sample when extracted in 150 μl). Of the 151 below the limit of detection, 39.2% (109/278) occurred in the samples collected during the day or evening and 56.0% (42/75) occurred among the samples collected in-bed at night. As noted previously, samples below the limit of detection were attributed with a value of half the detection limit, to allow the natural logarithm values of the exposure patterns to be determined. The total quantity (pg) of allergen sampled over the approximately 24 hour total period for each person ranged from 49.70 pg to 1301.94 pg with a geometric mean 238.91 pg. A distribution plot of the exposures (pg/m^3^) for the bed and non-bed samples is shown in S1 Fig.

The contribution of the bed can be estimated in two ways. When examined as separate 24 hour collections, the quantity of allergen collected in-bed-sleep samples, as a percent of the quantity collected over the ~24 hours, ranged from a minimum of 0.42% to a maximum of 34.19%, (an 80 fold difference) with a geometric mean of 5.55%. Alternatively, when the total quantity of allergen collected in all in-bed-sleep samples (category 1) was expressed as a percentage of the sum of all categories (1–8), this indicated that in-bed-sleep contributes 9.85% of total mite allergen exposure, Table 1. These estimates vary because of their distribution and the effects of having a considerable number of samples below the limit of detection. When the overnight in-bed sample was divided into 4 segments (first 30 minutes, last 30 minutes and the remainder into two equal portions of approximately 3 hrs each), the greatest exposure occurred in the first sample in 8 of the 11 instances where at least one of four samples was within the detection limit.

**Table 1:** The relative quantities and rates of exposure in the eight categories of location encountered in the 24 hour period.

No	Name of Category	Occurrence of each category (n)	Rate of exposure, pg/m^3^ (+/- 95% CI)	Geometric mean quantity of allergen collected in each category as a ratio of geometric mean of all other categories		Contribution of each category as a percentage of the total for the study	
				Ratio (+/- 95% CI)	p value #	Time (%)	Quantity of allergen (%)
1	Bedroom, sleeping	75	16.39 (9.42–28.54)	0.35 (0.24–0.52)	0.000	30.40	9.85
2	Bedroom, not-sleeping	43	66.21 (34.56–126.83)	1.91 (1.13–3.22)	0.015	7.70	7.83
3	House, not bedroom	136	71.59 (41.48–123.57)	2.59 (1.84–3.664)	0.000	25.99	41.86
4	Workplace/ Occupational	63	29.64 (16.604–52.904)	0.74 (0.47–1.18)	0.206	12.99	12.33
5	Public Building	18	38.12 (16.13–90.08)	1.02 (0.47–2.23)	0.952	4.25	4.13
6	Public Transport (Bus/Train/Ferry/Taxi)	13	87.80 (32.22–239.25)	2.48 (0.95–6.45)	0.062	1.87	4.18
7	Private Transport (Car)	36	44.77 (22.647–88.52)	1.23 (0.69–2.17)	0.480	4.63	4.73
8	Outdoors, and outdoor transport	79	47.01 (27.421–80.58)	1.36 (0.90–2.05)	0.141	12.19	15.08

^#^ p value from linear mixed regression model

The table shows a brief description of categories of location, numbered 1–8, the occurrence of each category among the 353 samples, the geometric mean (+/- 95% confidence interval) rate of exposure (pg/m^3^) of samples in that category, the ratio of the geometric mean quantity of exposure (pg) in that category as a function of the geometric mean of the sum of other categories (+/- 95% confidence intervals, and p values), and the contribution of the amount of time or quantity of allergen in each category expressed as a percentage of the sum of time or amounts of allergen for all categories. Note: 26.4% of samples involved more than one category of activity.

**Fig 2:**
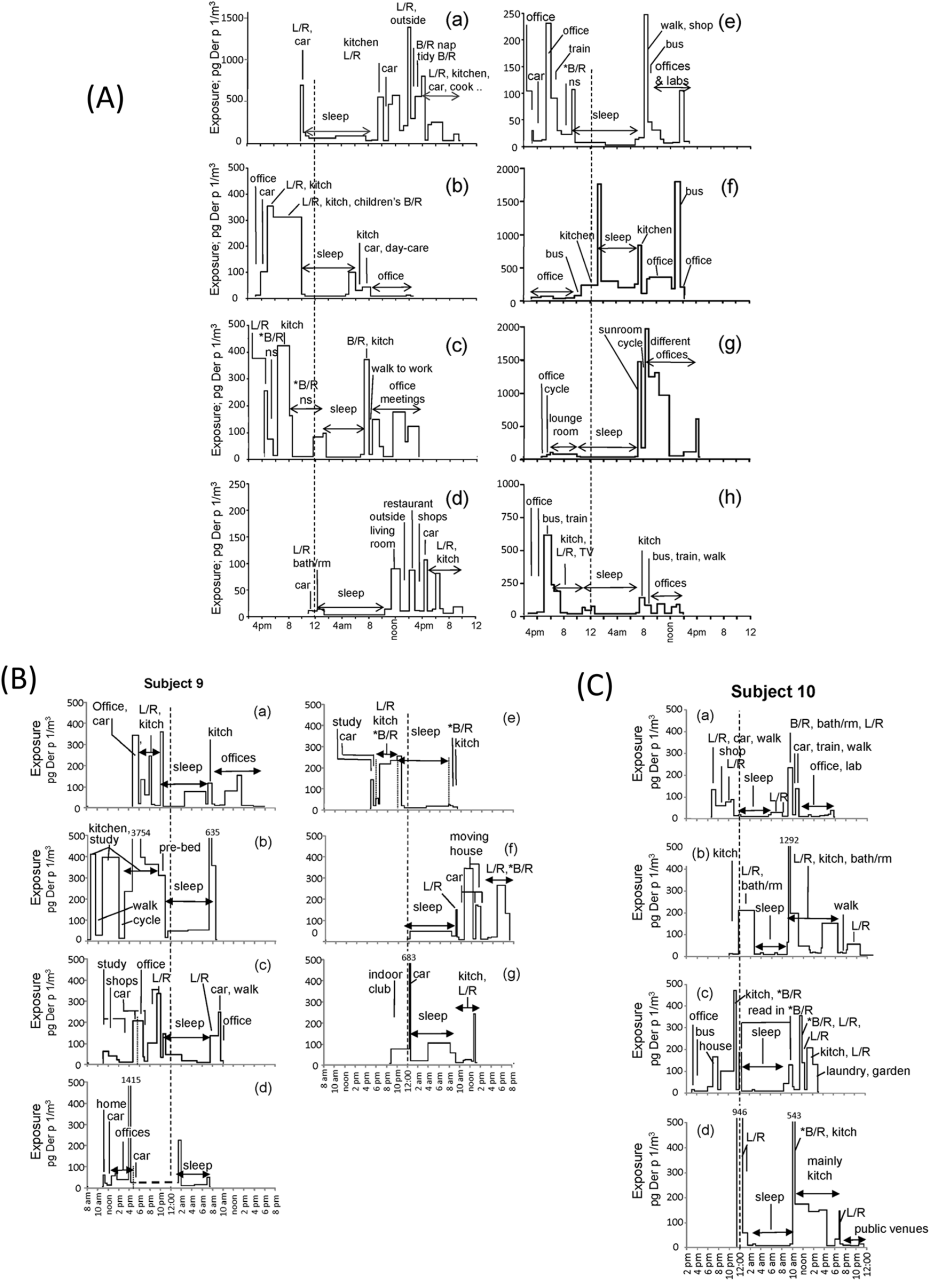
Plots of participant’s average exposures at discrete intervals over approximately 24 hours. The times of sampling have been aligned; midnight is shown as the vertical heavy dotted line. A lighter dotted line is used to separate samples that had similar quantities of exposure. The plots are annotated to nominate some of the activities or places. Where an average exposure exceeded the Y axis, it is denoted as a number above the gap in the plotted line. (A) The exposures of participants number 1–8 who collected samples over a single day and night period. The asterisk, i.e., ‘*B/R’ indicates that the person was in the bedroom but not sleeping, e.g. on computer, reading etc. Note different participants have a Y axis (exposure) maxima varying from 250–2000 pg/m^3^. (B) The exposures during eight collection periods by participant 9. Two part-days are shown on a single graph separated by a horizontal line; a third part-day is not shown. (C) The exposures during four collections made by participant 10. Both (B) and (C) use a maximum value on the Y axis of 500 pg/m^3^. Additional details of locations are tabulated in S1 Table.

Plots of the daily patterns of average exposure for each segment over the approximately 24 hour periods for the eight people who collected samples over a single day and night are shown in Fig 2A, and multiple samples by two people in Fig 2B and 2C respectively. These figures are annotated to identify some of the different activities and periods of sleep.

The geometric mean exposure (pg/m^3^) for each of these categories and the relative ratio of quantities associated with each category compared to all the other categories combined, an estimate of the percent contribution of category to the total exposure of the cohort, and the percent of time occupied by that activity, are all shown in Table 1. Only three activities were significantly different; the time in bed sleeping was significantly lower, whereas the time in the bedroom when not sleeping and the time around the house but not in the bedroom were both higher. Non-sleeping time in the bedroom included working on computers, relaxing and reading. The overall time in the house contributed 59.5% of the total exposure, and while public transport had the highest rate of exposure (geometric mean 87.8 pg/m^3^), this was infrequent and brief and its overall contribution to the total quantity was only 4.18%.

## Discussion

An important methodological limitation of current epidemiological studies is the lack of methods to measure the patterns of personal exposure to bioaerosols as a person moves unpredictably between different exposure-microenvironments over time. This poses particular novel technical challenges in the case of allergen exposure, as the concentration in the air fluctuates spatially over the small distances surrounding a person and temporally as the person moves within different locations or engages in different activities in the same location. In addition currently there are no methods to measure allergen in real-time. Further, exposure occurs as small, variable and discrete quanta at an airborne concentration of allergen per cubic metre, which often lies close to the limits of detection of conventional immunoassays. Our approach combines the use of a small personally-worn time-resolved impaction sampler, the use of a co-located miniature camera taking time-lapse images to record the location of the subject and the retrospective analysis of samples associated with categories of locations using a highly amplified ELISA.

The use and design of our in-house time-based impaction sampler had both strengths and limitations. The main advantages were that it allowed continuous monitoring of personal exposure during normal daily activity without the need for direct technical input of the participant, other than to change the disk once over the 24 hour period if the 12 hour clock was used and to cover the camera lens when recording was not socially appropriate. Secondly, it allows for separate subsamples to be selected for analysis following a post-hoc review of the time-lapse video. This means that samples corresponding to a single activity but of different lengths of time can be isolated, limiting the need to resolve samples reflecting multiple activities by modelling. This also maximises the detection in single samples where analyte concentrations are close to the detection limit of detection. A limitation with the current design is that the smallest segments that can be cut from the 25 mm disks corresponds to ~1.5 mm of the periphery of the sample disk, providing a resolution of between 15 to 30 minutes depending on the whether a 12 or 24 hours clock motor was used. This also means that there is some overlapping of particles impacted at times either side of the cut-line that defines each segment; while the total exposure remains the same, their resolution is compromised. A second limitation is that collection by impaction invokes a particle cut-off size below which particles are less efficiently collected. In our case the 50% cut-off was calculated to be 3.5 μm. Under conditions of personal exposure at least during the day, the large majority of mite, cockroach and cat allergens are carried on particles >3.5 μm [15–17].

Impaction samplers are typically subject to the loss of a small fraction of the sample onto the interior walls of the sampler (wall losses) as opposed to the collection surface. We found in preliminary studies that the mean total loss of approximately 40% was similarly distributed between the inlet nozzle, the interior wall surfaces and a fraction that was collected on a back-up filter. Lundgren reported average wall losses alone of four multi-stage impaction samplers as between 4.35 and 25% (maximum 51.9% for one stage) depending on the sampler [18]; this is not dissimilar to ours.

The absolute efficiency of the sampler, that is, the relationship of the amount of allergen collected by the sampler to the allergen concentration in the air was not established. The quantities sampled therefore reflect their relative proportions between categories, not the absolute amounts involved in human exposure.

Collection efficiency is also a function of retention by the impaction surface and both the electret and the i-Phone cover adhesive appear to be high and similarly efficient. Both are also novel for this purpose. There are hundreds of types of electrets, differing in thickness, fibre size, density and other characteristics. EWE50 was chosen based on our prior selection for collecting airborne virus for PCRs [19] and because it is thin with a fine porous texture. Electret may have advantages as an impaction surface due to both its porous surface [20] and its electrostatic attraction of particles [21] however this remains to be explored. We chose the i-Phone cover adhesive after a comparison of adhesives. We did not use the acrylic adhesives as used in our halogen assays [22] as they permanently bound to the protein binding membrane and this arrangement has a higher background signal than press-blotting. We could not reliably replicate results with other adhesives reported to have been used in press-blotting [23–25]. We found allergens could either be press-blotted or eluted from the iPhone adhesive. Again, this impaction surface is novel and further evaluation remains to be performed.

Clearly many design variants of such a personal sampler are possible. Originally, we chose 25 mm for the diameter of the disks as we anticipated performing a single analysis on the whole disc, using a combination of press-blotting, immuno-chemiluminescence and image analysis (that is, the disk would not be cut into wedges) and 25 mm is a common diameter for protein binding membranes. However, in the pre-pilot development phase this detection method was not as quantitative as the highly amplified ELISA. For illustration, some examples are included in S1 Text, without a full description of the methods or results. If the resolution of time is important, and it generally is, then a larger diameter disk should be used. Subsequent to these experiments the sampler has been redesigned to use 50 mm membranes (or 10 mm wide strips around the circumference edge of a 50 mm disk) and a narrower slot. This should provide a resolution of less than 8 minutes with a 12-hour clock, again see S1 3D File for such a design. A useful modification would be to use a programmable stepper motor in place of the clock motor, so that both the total sample time, and interval between discreet (non-overlapping) samples could be controlled, depending on the sampling situation.

The only other study we are aware of to continuously collect domestic allergen exposure using time-based sampling methods used a semi-portable device (about the size of a small shoe box) and detected the allergens using immunostaining [25]. A more recent study of allergen and endotoxin exposure over the day and night used a combination of static and personal samplers to provide a coarse resolution of exposures in locations at home, school and bedroom [9]. While the main purpose of this other study was to quantify the re-suspension of bioaerosols, their limited results showing lower exposures in the bedroom are consistent with ours.

The use of time-lapse monitoring enables the activities and place to be easily identified with high resolution without inaccuracies and compliance issues inherent in personal record keeping. Because individual images were sometimes compromised, we found it preferable to sample at 15 second intervals rather than less frequently. We also acknowledge that taking covert images in public and domestic locations potentially raises issues of privacy and consent; participants were informed about this and counselled to cover the camera in situations where taking such images may be discourteous or invasive, however this remains an issue of sensitivity and full disclosure, permission and caution is required. It is not necessary to photograph the other people present; images of the floor often allow the location to be identified.

Overall, while around 60% of total exposure occurred in the home at different times during the day and evening, the samples collected in-bed-asleep only contributed around 5–10% of total exposure on average, depending on how this was estimated. Because of the small sample size, such a conclusion should be regarded only as indicative and not be over-generalised. However, these results do support an emerging model of mite aeroallergen exposure which is at variance with an earlier convention that the bed is the main site of exposure. Exposure in bed was highest on bed entry and is typically lower for most of the night when there is little movement despite the bed dust containing high concentrations of allergen. These current results are also consistent with our earlier study using different methods to indicate the influence of people, activity and clothing as sources of personal mite allergen exposure over the 24 hour period [11] and the study of exposure of US school children [9].

These studies demonstrate that personal exposure varies greatly both over time within subjects and also between subjects. The reasons for the variations presumably reflect the complex dynamics of particles being aerosolised from different sources and by different activities. Local allergen reservoirs would include the clothing of both the participant and other people in their vicinity, as well as from furnishing and carpets, all of which would differ in source strength. Aerosolisation from these would occur with human movement and other forms of mechanical disturbance. Additional factors such as body thermals, and removal of particles by settling and ventilation would further modify these exposures. We and others have shown clothing is a potent source of domestic aeroallergens [10, 26, 27], and such dust is disturbed by body movement, to create ‘personal clouds’ of bioaerosol exposures [28].

This study has several other limitations in terms of describing a generalised pattern of mite exposure; the number of participants is very small and they are of a similar demographic; most were only sampled once, although two were sampled repeatedly and in these samples exposure varied between days. We would also expect such findings to differ with other social groups, allergens, geographies, or lifestyles as well as with season, recent domestic hygiene practices and many other factors.

It is also apparent that for any person, there can be a wide variation in exposures during the course of the day even at a single location such as in an office or in the house. This probably demonstrates the importance of the activity of the person and the presence of other people contributing personal aerosols or to dust disturbance. For simplicity, we only assigned eight categories to location, including only three domestic. People often moved fluidly between the different living (non-bedroom) areas of the house and these were clustered together; whereas for the bedroom it was necessary to differentiate between the times of sleeping at night and the times of working on a computer or reading in the bedroom during the day. Other non-domestic locations were selected to emphasise that mite exposure occurs in occupational and public locations and during transport. Information on locations is provided in the annotations of Fig 2A–2C and in more detail in S1 Table.

This form of collection only approximates what is actually inhaled. Although the sampler is located close to the breathing zone, much aeroallergen exposure probably occurs as small, local ‘clouds’ of particles of different sizes that carry different and discrete ‘quanta’ of allergens [29, 30]. The chance of encounters with such particles is the subject of probabilities and is modified by local air currents and thermals [31, 32]. Within this study there is also likely to be relative differences in what is collected by the sampler when it is located close to the pillow when the person is lying immobile in bed, compared to when the sampler is on the lapel and the person is upright and active [4, 33, 34]. While nocturnal exposure may be relatively underestimated in this study compared to that in the day, the overall quantity is still probably relatively low, given that nearly 60% of nocturnal samples were below the detection limit, despite three hour sampling times. When detectable exposures occurred in bed, this was mainly during the 30 minute sample collected on bed entry, not during the night itself. It is important to further clarify this with additional studies.

Our results have practical clinical implications. The prevalence of allergic disease affects close to the majority of some populations and these rates are increasing. Allergen exposure probably plays a significant causal role in the symptoms of perennial rhinitis [35] and both a causal and synergistic role together with other factors in exacerbations of asthma [36]. However, the use of environmental interventions is not supported by many treatment guidelines due to meta-analyses of such trials often failing to find consistent clinical benefits [2]. Why such interventions have failed is likely to be at least in part because they were largely directed at beds, and, as our results demonstrate, most exposure occurred elsewhere. Additionally, the rationale for interventions based on bed encasings was largely based on reservoir measurements, but encasings themselves quickly became contaminated and themselves act as allergen sources; a factor not evident unless aeroallergen is measured [37]. Isolated studies that used multi-facetted or whole-house approaches to reducing exposure [38–40] or supplied ultraclean air to the bed airspace [41] have shown more success. Measuring such patterns of exposure will provide insight into the sources and events associated with high exposure and provide better indices to associate with clinical outcomes and allow the development of targeting of interventions to effectively reduce exposure.

It is realistic to propose that in future clinically-symptomatic individuals could use these methods to continuously monitor their exposure to one or more allergens (or to all personally-relevant allergens using their own IgE for analysis [22]) over several days and determine their sources of greatest exposure. This would be a powerful, novel and individual approach to managing allergic diseases that has not been available in the clinical armamentarium.

In conclusion, an important methodological limitation of current epidemiological studies has been the inability to describe the ‘time-geography’ of personal exposure to bioaerosols over extended periods of time. Here we report our pilot studies which show the feasibility of combining time-lapse photography and time-based collection of personal exposures to quantify the patterns of mite allergen exposures occurring over 24 hour periods. The methods have wide application for other situations (occupational, industrial etc), to other bioaerosols (fungi, endotoxin, microflora, viral aerosols etc) and to other forms of analysis (PCR, direct image analysis, Halogen, chemiluminescence etc) which can be used with these or similar collection surfaces. Our data provides insight into the association of mite allergen exposure with place and activity. Further they provide a basis to both speculate on why allergen avoidance interventions might have failed in the past and how to design better strategies for the future. In particular, for the first time, individuals were able to identify the important sites of their own exposure.

## Supporting Information

S1 Fig Distribution of aeroallergen exposure in bed and non-bed samples. Frequency of occurrence (%) is shown on the Y axis and exposure (pg/M^3^) using a log scale on the X axis. Because a value of half the assay detection limit was attributed to samples below this limit, and because the sub-samples had different collection times, there was a variation in the apparent exposure for samples below the detection limit (dotted lines). Exposures for samples above the assay detection limit are shown as solid lines. Samples collected while in bed (blue line) were collected in the four periods of sleep: 30 mins after entering bed, 30 minutes before getting out of bed, and the remaindering time in the night, divided into equal portions of approximately 3 hours each. All other samples were collectively called ‘non-bed’ samples, (red line); this would include samples collected outside, inside the house and including in the bed room during the day when the subject was reading, using a computer, watching TV. There was a statistically significant difference between the two groups (P = 0.0177) above the detection limit when compared using a two-sample t-test with equal variances of the natural logs of the values. On the log scale, values from those in the non-bed group (log mean = 4.14) are 0.6083 larger than those from the bed (log mean = 4.7492). Overall, the non-bed group are 1.84 times (95%CI = +/-3.033145) larger than the bed group.
(TIF)

S1 File 3-D printer files of samplers. The folder contains zipped 3D printer files for the original sampler used these experiments. This consists of the plate fitting to the front of the clock body containing the inlet slot and air outlet, plus files for the disks which fitted onto the hour hand shaft of the clock. Additionally, 3D files for a second untested design are supplied, modified by the experience with the original sampler.
(ZIP)

S1 Table Raw data files for sampling, categories and sampler performance. Excel files of the data set for all samples, their sampling time, allergen content, location category and a brief description of locations. Additionally, files for the results comparing the efficiency of the two sampling surfaces, the losses to the walls and orifice, and the data dictionary for the samples.
(XLSX)

S1 Text A summary of experience with press-blotting and chemiluminescent detection of allergen. This alternative method of performance used press blotting allergen from the entire adhesive disk surface using membranes containing allergen-specific antibodies and subsequent assay using a second specific antibody, followed by detection using chemiluminescent imaging. This method has some advantages, but requires more technical development.
(DOC)

## References

[1] Pawankar R, Canonica G, Holgate S, Lockey R. Blaiss, MS World Health Organization. White Book on Allergy Update 2013, 2013. Available: http://www.worldallergy.org/UserFiles/file/WhiteBook2-2013-v8.pdf

[2] Gotzsche PC, Johansen HK. House dust mite control measures for asthma. Cochrane Database of Systematic Reviews. 2008 10.1002/14651858.CD001187.pub3 ISI:000255119900087. PMC8786269 18425868

[3] Tovey ER, Marks GB. It's time to rethink mite allergen avoidance. J Allergy Clin Immunol. 2011;128:723–727. 10.1016/j.jaci.2011.07.009 . 21855978

[4] Gore RB, Boyle RJ, Gore C, Custovic A, Hanna H, Svensson P, et al Effect of a novel temperature-controlled laminar airflow device on personal breathing zone aeroallergen exposure. Indoor Air. 2015;25:36–44. 10.1111/ina.12122 24750266

[5] Morawska L, Afshari A, Bae GN, Buonanno G, Chao CYH, Hanninen O, et al Indoor aerosols: from personal exposure to risk assessment. Indoor Air. 2013;23:462–487. 10.1111/ina.12044 . 23574389

[6] Bhangar S, Huffman JA, Nazaroff WW. Size-resolved fluorescent biological aerosol particle concentrations and occupant emissions in a university classroom. Indoor Air. 2014;24:604–617. 10.1111/ina.12111 . 24654966

[7] Tian Y, Sul K, Qian J, Mondal S, Ferro AR. A comparative study of walking-induced dust resuspension using a consistent test mechanism. Indoor Air. 2014;24:592–603. 10.1111/ina.12107 . 24605758

[8] Tovey E, De Lucca SD, Pavlicek P, Sercombe J, Taylor D, O'Meara T. The morphology of particles carrying mite, dog, cockroach and cat aeroallergens affects their efficiency of collection by nasal samplers and cascade impactors. J Allergy Clin Immunol. 2000;105:S228, Abstract 676. 10.1016/S0091-6749(00)91104-7

[9] Raja S, Xu Y, Ferro AR, Jaques PA, Hopke PK. Resuspension of indoor aeroallergens and relationship to lung inflammation in asthmatic children. Environ Int. 2010;36:8–14. 10.1016/j.envint.2009.09.001 19796820

[10] Karlsson AS, Andersson B, Renstrom A, Svedmyr J, Larsson K, Borres MP. Airborne cat allergen reduction in classrooms that use special school clothing or ban pet ownership. J Allergy Clin Immunol. 2004;113:1172–1177. 10.1016/j.jaci.2003.12.590 15208601

[11] Tovey ER, Willenborg CM, Crisafulli DA, Rimmer J, Marks GB. Most Personal Exposure to House Dust Mite Aeroallergen Occurs during the Day. PloS One. 2013;8(7) e69900 10.1371/journal.pone.0069900 . 23894558 PMC3722239

[12] Graham J, Pavlicek P, Sercombe J, Xavier M, Tovey E. The nasal air sampler. A device for sampling inhaled aeroallergens. Ann Allergy Asthma Immunol. 2000;84:599–604. 10875488 10.1016/s1081-1206(10)62410-6

[13] Marple VA, Willeke K. Impactor design. Atmospheric Environ. 1976;10:891–896.

[14] Glasgow NJ, Ponsonby A-L, Kemp A, Tovey E, van Asperen P, McKay K, et al Feather bedding and childhood asthma associated with house dust mite sensitisation: a randomised controlled trial. Arch Dis Child. 2011;96:541–547. 10.1136/adc.2010.189696 21451166 PMC3093241

[15] Montoya LD, Hildemann LM. Size distributions and height variations of airborne particulate matter and cat allergen indoors immediately following dust-disturbing activities. J Aerosol Sci. 2005;36:735–749. 10.1016/j.jaerosci.2004.11.004 ISI:000229348600014.

[16] Tovey ER, Chapman MD, Wells CW, Platts-Mills TA. The distribution of dust mite allergen in the houses of patients with asthma. Am Rev Respir Dis. 1981;124:630–635. . 7305119 10.1164/arrd.1981.124.5.630

[17] De Lucca S, Taylor D, O'Meara T, Jones A, Tovey E. Measurement and characterization of cockroach allergens detected during normal domestic activity. J Allergy Clin Immunol. 1999;104:672–680. 10482845 10.1016/s0091-6749(99)70341-6

[18] Lundgren DA. An aerosol sampler for determination of particle concentration as a function of size and time. J Air Pollut Control Assoc. 1967;17:225–259. . 6038467 10.1080/00022470.1967.10468972

[19] Tovey ER, Stelzer-Braid S, Toelle BG, Oliver BG, Reddel HK, Willenborg CM, et al Rhinoviruses significantly affect day-to-day respiratory symptoms of children with asthma. J Allergy Clin Immunol. 2015;135:663–669. 10.1016/j.jaci.2014.10.020 25476729 PMC7173323

[20] Lee SJ, Demokritou P, Koutrakis P. Performance evaluation of commonly used impaction substrates under various loading conditions. J Aerosol Sci. 2005;36:881–895. 10.1016/j.jaerosci.2004.11.006 WOS:000230367200005.

[21] Rengasamy S, Eimer BC, Shaffer RE. Comparison of Nanoparticle Filtration Performance of NIOSH-approved and CE-Marked Particulate Filtering Facepiece Respirators. Ann Occup Hyg. 2009;53:117–128. 10.1093/annhyg/men086 . 19261695

[22] Tovey E, De Lucca S, Poulos L, O'Meara T. The Halogen assay—A new technique for measuring airborne allergen. Methods Mol Med. 2008;138:227–246 10.1007/978-1-59745-366-0_19 18612612

[23] Schumacher MJ, Griffith RD, O'Rourke MK. Recognition of pollen and other particulate aeroantigens by immunoblot microscopy. J Allergy Clin Immunol 1988;82:608–616. 3049747 10.1016/0091-6749(88)90972-4

[24] Takahashi Y, Nagoya T, Watanabe M, Inouye S, Sakaguchi M, Katagiri S. A new method of counting airborne Japanese cedar (Cryptomeria japonica) pollen allergens by immunoblotting. Allergy. 1993;48:94–98. 8457039 10.1111/j.1398-9995.1993.tb00692.x

[25] Sakaguchi M, Inouye S, Takahashi Y, Katagiri S, Yasueda H. Immunoblotting of mite aeroallergens collected with an indoor Burkard air sampler. Aerobiologia. 1995;11 265–268. 10.1007/BF02447207

[26] De Lucca SD, O'Meara TJ, Tovey ER. Exposure to mite and cat allergens on a range of clothing items at home and the transfer of cat allergen in the workplace. J Allergy Clin Immunol. 2000;106:874–879. . 11080709 10.1067/mai.2000.110804

[27] Patchett K, Lewis S, Crane J, Fitzharris P. Cat allergen (Fel d 1) levels on school children's clothing and in primary school classrooms in Wellington, New Zealand. J Allergy Clin Immunol. 1997;100:755–759. . 9438482 10.1016/s0091-6749(97)70269-0

[28] Rabinovitch N, Liu AH, Zhang LN, Rodes CE, Foarde K, Dutton SJ, et al Importance of the personal endotoxin cloud in school-age children with asthma. J Allergy Clin Immunol. 2005;116:1053–1057. 10.1016/j.jaci.2005.08.045 16275375

[29] Doekes G, Thorne PS, Sander I, Wouters I, Eduard W, Heederik D. Airborne Allergen Exposure As a Quantum Phenomenon. J Allergy Clin Immunol. 2014;133:Abs 187. ISSN: 0091-6749 WOS:000330241300642.

[30] De Lucca S, Sporik R, O'Meara T, Tovey ER. Mite allergen (Der p 1) is not only carried on mite feces. J Allergy Clin Immunol. 1999;103:174–175. 9893203 10.1016/s0091-6749(99)70543-9

[31] Spilak MP, Boor BE, Novoselac A, Corsi RL. Impact of bedding arrangements, pillows, and blankets on particle resuspension in the sleep microenvironment. Build Environ. 2014;81:60–68. 10.1016/j.buildenv.2014.06.010 WOS:000342532200008.

[32] Liden G, Surakka J. A Headset-Mounted Mini Sampler for Measuring Exposure to Welding Aerosol in the Breathing Zone. Ann Occup Hyg. 2009;53:99–116. 10.1093/annhyg/mep001 19196747

[33] Lau JY, Sercombe JK, Tovey ER Characterisation of personal Der p 1 exposure from upper bedding, when placed in a clean environment. J Allergy Clin Immun. 2005;115:S94 Abs 378. 10.1016/j.jaci.2004.12.390

[34] Gore R, Boyle RJ, Hanna H, Custovic A, Gore C, Svensson P, et al Personal allergen exposure is increased by turning over in bed and improved by temperature-controlled laminar airflow. Clin Exp Allergy. 2011;41:1848–1848. WOS:000297283800097. ISSN: 0954-7894

[35] Klimek L, Sperl A, Raulf M. Allergic rhinitis to mites—workup and treatment. Allergologie. 2015;38:70–82. WOS:000350393100005.

[36] Murray CS, Poletti G, Kebadze T, Morris J, Woodcock A, Johnston SL, et al Study of modifiable risk factors for asthma exacerbations: virus infection and allergen exposure increase the risk of asthma hospital admissions in children. Thorax. 2006;61:376–382. 10.1136/thx.2005.042523 16384881 PMC2111190

[37] Lau JY, Sercombe JK, Rimmer JS, Tovey ER. Bed encasings reduce reservoir allergens but not aeroallergens. J Allergy Clin Immun. 2006;117:S30, Abs 120. 10.1016/j.jaci.2005.12.125 WOS:000235865300120

[38] Xu Y, Raja S, Ferro AR, Jaques PA, Hopke PK, Gressani C, et al Effectiveness of heating, ventilation and air conditioning system with HEPA filter unit on indoor air quality and asthmatic children's health. Build Environ 2010;45:330–337. 10.1016/j.buildenv.2009.06.010 WOS:000271970000010

[39] Scott M, Roberts G, Kurukulaaratchy RJ, Matthews S, Nove A, Arshad SH. Multifaceted allergen avoidance during infancy reduces asthma during childhood with the effect persisting until age 18 years. Thorax. 2012;67:1046–1051. 10.1136/thoraxjnl-2012-202150 22858926

[40] Krieger J. Home Is Where the Triggers Are: Increasing Asthma Control by Improving the Home Environment. Pediatr Asthma Immu Pulm. 2010;23:139–145. 10.1089/ped.2010.0022 . 22375276 PMC3281289

[41] Boyle R, Pedroletti C, Wickman M, Bjermer L, Valovirta E, Dahl R, et al Nocturnal temperature controlled laminar airflow for treating atopic asthma: a randomised controlled trial. Thorax. 2012;67:215–221. 10.1136/thoraxjnl-2011-200665 22131290 PMC3282042

