# Supplementary figures and images for: Time-Based Measurement of Personal Mite Allergen Bioaerosol Exposure over 24 Hour Periods
Source: PLoS One. 2016 May 18;11(5):e0153414. doi: 10.1371/journal.pone.0153414 (PMC4871444; doi:10.1371/journal.pone.0153414)

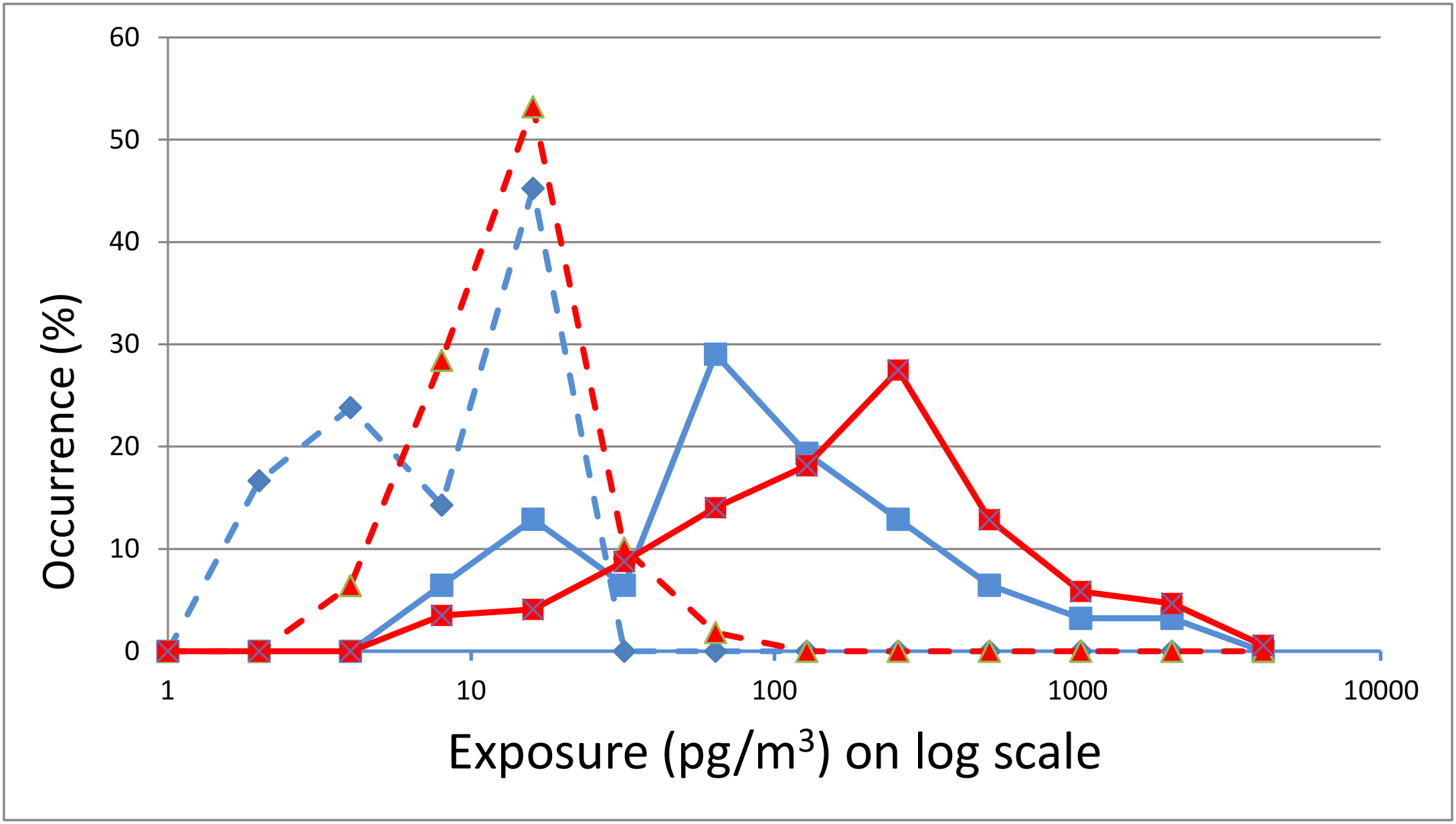

Supplement: S1 Fig — Frequency of occurrence (%) is shown on the Y axis and exposure (pg/M3) using a log scale on the X axis. Because a value of half the assay detection limit was attributed to samples below this limit, and because the sub-samples had different collection times, there was a variation in the apparent exposure for samples below the detection limit (dotted lines). Exposures for samples above the assay detection limit are shown as solid lines. Samples collected while in bed (blue line) were collected in the four periods of sleep: 30 mins after entering bed, 30 minutes before getting out of bed, and the remaindering time in the night, divided into equal portions of approximately 3 hours each. All other samples were collectively called ‘non-bed’ samples, (red line); this would include samples collected outside, inside the house and including in the bed room during the day when the subject was reading, using a computer, watching TV. There was a statistically significant difference between the two groups (P = 0.0177) above the detection limit when compared using a two-sample t-test with equal variances of the natural logs of the values. On the log scale, values from those in the non-bed group (log mean = 4.14) are 0.6083 larger than those from the bed (log mean = 4.7492). Overall, the non-bed group are 1.84 times (95%CI = +/-3.033145) larger than the bed group. (TIF) [file pone.0153414.s001.tif]
