# Supplementary material for: Time-Based Measurement of Personal Mite Allergen Bioaerosol Exposure over 24 Hour Periods
Source: PLoS One. 2016 May 18;11(5):e0153414. doi: 10.1371/journal.pone.0153414 (PMC4871444; doi:10.1371/journal.pone.0153414)
Supplement: S1 Text — This alternative method of performance used press blotting allergen from the entire adhesive disk surface using membranes containing allergen-specific antibodies and subsequent assay using a second specific antibody, followed by detection using chemiluminescent imaging. This method has some advantages, but requires more technical development. (DOC) [file pone.0153414.s004.doc]

**Summary of experience with press-blotting and chemiluminescent detection**

**Collection surface**

We found the most suitable impaction surface for collection of particles and analysis by chemiluminescence was the multilayered sheet used to protect the screens of i-Phones and smart devices. The protective disposable film is peeled off to expose a very thin layer of a gel-like adhesive film bound to the clear plastic sheet. This adhesive film was used as the impaction surface for sampling. In our experience it had high particle retention in collection and could easily be removed from the membrane without damaging the latter in blotting.

**Assays**

First a press-blotting membrane containing a capture antibody was prepared. Specifically a 25 mm diameter 0.45 µm pore size mixed cellulose ester membrane (Millipore HA) was incubated with a monoclonal antibody 5H8 from an ELISA kit for mite allergen Der p 1 (Indoor Biotechnology, Charlottesville, USA) at 1:1000 in carbonate/bicarbonate buffer overnight, and then blocked using 1% BSA in PBS (0.05% Tween 20) for 1 hour. The adhesive film with the captured particles was directly applied to the damp antibody-coated membrane for 40 minutes and then removed. The membrane was then incubated with biotinylated second antibody (4C1) from the ELISA kit at 1:1000 for 1 hr, washed and then incubated with Streptavidin peroxidase (1:2000) for 30 minutes, washed and the allergen detected using chemiluminescent detection (Millipore Immobilon Western Chemiluminescent HRP Substrate Kit, WBKLS0500, Millipore, Billerica, USA). The membrane was imaged on a Kodak Image Station (Model 4000mm) using standard Western Blotting protocols. All washing was performed as series comprising, 1, 5, 10 and 10 minute washes.


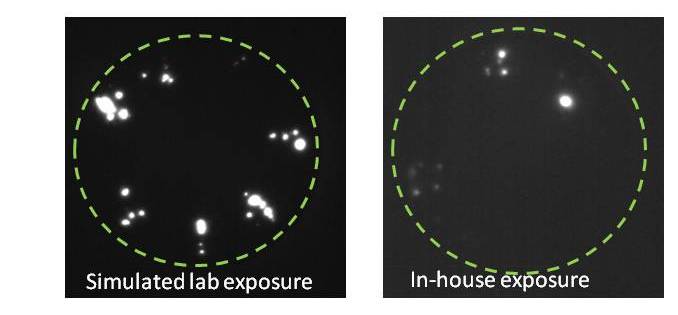


Figure S 1: Images of detection of mite allergens impacted around the periphery of an adhesive disk in the sampler by chemiluminescence following press-blotting. Figure on the left is from simulated mite exposure performed in the laboratory over 12 hours. The figure on the right is from collection in a house during a normal day. Adopting the nomenclature of a clock face, luminescent spot(s) are visible around the 2, 8-9 and 12 o’clock positions.

We found that using a capture system for the allergen (ie an antibody coated membrane) was superior to using a fresh (unblocked) protein binding membrane in terms of greater signal strength and lower background. Earlier studies of press-blotting have used an unblocked protein-binding membrane. However, we were unable to validate and quantify the amount of allergen represented by the individual spots of chemiluminescence. Specifically, we were unsuccessful in our attempts to print a set of known allergen standards onto the membrane to create a standard curve for interpretation of the quantities of allergen associated with each spot. Additionally, the use of standard imaging devices designed for measuring chemiluminescence from western blots may have limited our capacity to quantify the small spots of luminescence produced by allergen sources.

Analysis of the whole disk by chemiluminescent staining differs in several ways compared to analysis by cutting and extracting individual time-based wedges of the disk and assaying these by ELISA. The obvious advantage of the former is its simplicity in terms of analysis and handling of a single membrane, plus the greater time-based resolution of individual exposure events, even within the different categories of location. The use of wedges analysed by ELISA only provides the mean exposure over each period. Perhaps the most obvious subjective difference is that chemiluminescent staining visually demonstrates that allergen exposure involves particles, each carrying very different quantities of allergen. Because these encounters are uncommon, there is a significant variation in apparent exposure simply on the randomness of encountering them (or not) within that air sample. As noted we could not validate the quantification of allergen associated with the separate spots, but the subjective impression was that chemiluminescence was not as sensitive as ELISA in detecting periods of low exposure. More expertise, experience and dedicated instrumentation and programming may overcome these shortfalls. Quantified immuno-press-blotting of the entire sample remains our ideal for such a system due to its simplicity of handling, and the much greater precision in resolving the time of exposure events.
